# The Role of Myositis-Specific Autoantibodies in the Dermatomyositis Spectrum

**DOI:** 10.7759/cureus.22978

**Published:** 2022-03-08

**Authors:** Harshita Marasandra Ramesh, Sai Sreeya Gude, Shravya Venugopal, Nikhil Chowdary Peddi, Sai Sravya Gude, Sravya Vuppalapati

**Affiliations:** 1 Internal Medicine/Pediatrics, Kasturba Medical College, Mangalore, Mangalore, IND; 2 Internal Medicine, Guntur Medical College, Guntur, IND; 3 Internal Medicine, Kasturba Medical College, Mangalore, Mangalore, IND; 4 Pediatrics, PES Institute of Medical Sciences and Research, Kuppam, IND; 5 Pediatrics, Guntur Medical College, Guntur, IND; 6 Paediatrics, PES Institute of Medical Sciences and Research, Kuppam, IND

**Keywords:** populations genetics, auto immune, adult-onset dermatomyositis, juvenile dermatomyositis, auto antibodies

## Abstract

Dermatomyositis (DM) is a systemic autoimmune disease that affects skeletal muscles, the skin, and the lungs. It is characterized by autoantibodies, tissue inflammation, parenchymal cell damage, death, and vasculopathy. In terms of epidemiology, DM affects both children and adults. The current pathophysiology of DM is described as an autoimmune attack on the afflicted organs driven by environmental variables such as UV exposure, medications, infections, and lifestyle choices in genetically predisposed people. DM is also a paraneoplastic condition, which means that cancer may arise before, along with, or following the development of the symptoms of DM.

Myositis-specific autoantibodies are associated with phenotypical features and are used for sub-classification of dermatomyositis patients. Because the risk of interstitial lung disease (ILD), internal malignancy, destructive disease trajectory, and maybe a response to medication differs by DM myositis-specific antibody (MSA) group, a better knowledge of MSAs and the validation and standardization of tests employed for detection is crucial for improving diagnosis and treatment. The diagnostic sensitivity and specificity of tests for various MSAs are not ideal, just like with any other test. However, more antibody tests are anticipated to make their way into formal schemata for diagnosis and actionable risk assessment in DM due to worldwide standardization and more extensive research. In this review, we outline crucial aspects for interpreting clinical and pathologic relationships with MSA in DM and critical knowledge and practice gaps that will optimize the clinical benefit and utility of MSAs as diagnostic and prognostic markers.

## Introduction and background

Idiopathic inflammatory myopathies (IIMs) are a group of illnesses that affect the systemic skeletal muscles and are frequently associated with extramuscular complications such as interstitial lung disease (ILDs), arthritis, and cancer. Polymyositis (PM), dermatomyositis (DM), and inclusion myositis (IM) are three different types of myositis and are major subcategories of IIMs [1]. DM is a multifactorial, chronic autoimmune condition characterised by skin abnormalities and organ involvement, including the muscles, blood vessels, joints, esophagus, and lungs. A variety of intrinsic and extrinsic variables, including genetic, environmental, immunological, and nonimmune-mediated factors, have been implicated in illness etiology [2]. Juvenile-onset myositis is an extremely uncommon condition, with an incidence of 2-4 per million. Juvenile dermatomyositis (JDM) is commonly used as a catch-all term for all juvenile-onset myositis since the great majority of afflicted children have a cutaneous illness. There is tremendous variation within the JDM subgroup, with varying chronicity, organ involvement, and long-term clinical prognosis [3]. The Bohan and Peter criteria [4,5], proposed in 1975, have been widely used to identify patients; however, they do not adequately describe the full spectrum of myositis and do not provide a definition for clinically amyopathic myositis, immune-mediated necrotizing myositis (IMNM), or inclusion body myositis, which is not seen in children [3]. New categorization criteria for adult and adolescent idiopathic inflammatory myopathies and their major subtypes were released in 2017 by the European League Against Rheumatism and the American College of Rheumatology. The new criteria include limitations, such as excluding myositis-specific autoantibodies (excluding antiJo-1) and histological characteristics related to IMNM [6].

IIMs have incidence rates of 2.47-7.8 per 100,000 person-years and prevalence rates of 9.54-32.74 per 100,000 people, according to epidemiological research. In the United States, the prevalence of DM has been estimated at 1-6 per 100,000 people. DM is the most frequent of the IIMs, with DM being identified in 31% of patients in a recent examination of 3,067 individuals in the Euromyositis registry. DM affects both men and women, with a female to male ratio of 2:1. All ethnic groups are impacted, but African Americans are the most afflicted [7]. JDM is most often diagnosed between the ages of 4 and 14, whereas adult dermatomyositis (ADDM) is most typically diagnosed between the ages of 40 and 60. With an estimated frequency of 3.2 cases per million children per year, JDM is the most frequent inflammatory myopathy in children. The prevalence of clinically amyopathic JDM (CAJDM) is unknown. A recent study found that 25% of CAJDM patients subsequently acquire muscular involvement [7].

This review article will provide an in-depth view of dermatomyositis, pathogenesis, antibodies involved, clinical presentation, diagnosis, prognosis, and future prospects of these antibodies.

## Review

Pathogenesis

The pathophysiology of DM is complicated, complex, and poorly understood. Both adult DM and JDM development are assumed to be influenced by genetic, environmental, and immunological processes [7]. On a biological level, CD4+ T cells, B cells, and dendritic cells make up the inflammatory infiltration in DM muscle tissue, which is largely concentrated around perimysial blood vessels and perifascicular sites. Variable infiltration of CD4+ T cells, dendritic cells, and mast cells has been seen in DM skin. This inflammation is thought to cause damage to the parenchyma and blood vessels, resulting in the histologic appearance of perimysial and perifascicular atrophy in muscle specimens and keratinocyte injury (lichenoid tissue response) in the skin [8].

Genetic Factors

There is a major hereditary component to DM. Multiple genotyping investigations have found links between major histocompatibility complex (MHC) variants and the development of DM, and certain human leukocyte antigen (HLA) alleles have been linked to the generation of autoantibodies in both adults and children. In addition, in genome-wide investigations of juvenile IIMs, the International Genetics Consortium in Myositis (MYOGEN) discovered cytokine and lymphocyte signaling alleles linked to disease onset, severity, calcinosis, and ulceration. DNA methylation, histone modification, and miRNA activity may all have a role in the etiology of DM [7].

Environmental Factors

In genetically vulnerable people, a variety of environmental events can cause persistent immunological activation. UV light, viral infections, medicines, and smoking have all been proposed as DM causes. Adult women have been associated with DM and anti-Mi2 antibodies, whereas children have been connected to JDM and anti-transcription intermediary factor-1 (anti-TIF-1) antibodies. Viral infections may have a role in immunological activation or immune tolerance disruption [7].

Immunological Factors

The exact sequence of immunological activation in DM is unknown; however, it is thought to be caused by excessive complement activation. It is still up for debate whether this activation is antibody-dependent or stems from the start of the conventional complement cascade. Regardless, capillary damage results in ischemia and microinfarction, hypoperfusion, and perifascicular atrophy as a result of this activation. In addition to defective cell differentiation and maturation, altered expression of myogenic regulatory factors (as seen in JDM) may lead to atrophy [7].

According to research, interferons (IFN) are also thought to have a role in DM and JDM. The type I IFN pathway has been found to be upregulated in the muscle, skin, and blood of diabetic patients, and cutaneous activity in adult DM has been linked to a type I IFN gene signature [9-13].

Type I IFN score, type II IFN score, and tumor necrosis factor (TNF) alpha expression are all linked to disease activity in JDM patients. Multiple autoimmune illnesses have been linked to a prolonged IFN response (caused by chronic stimulation of antigen-presenting cells); the ensuing T and B cell activation may be responsible for auto-antibody formation. However, the pathogenic role of these auto-antibodies in DM and JDM is unknown. Anti-endothelial cell antibodies have recently been discovered in the plasma of children with JDM, confirming that JDM is an antibody-mediated vasculopathy [7].

Clinical features in classic DM

DM is marked by a variety of muscle and cutaneous symptoms as well as consequences such as ILD and malignancy [14-17]. The variability of DM makes diagnosis difficult [18]. Other challenges in diagnosis are assessing the patient for occult malignancy, other orang involvement, and similar clinical presentations to autoimmune rheumatologic disorders like systemic lupus erythematosus, mixed connective tissue disorder, rheumatoid arthritis, cutaneous lupus erythematosus, inflammatory myopathies, and cutaneous eruptions [18]. DM has a bimodal onset distribution of incidence: onset in childhood between 5 and 10 years of age and in adulthood between 40 and 60 years of age, with a female preponderance [17].

Cutaneous Manifestations

The cutaneous findings are categorized as pathognomonic, characteristic, compatible, and rare non-specific features [7,17].

Pathognomonic skin features include Gottron’s papule and Gottron’s sign. Gottron’s papules are elevated purplish lesions on an erythematous base. It is most commonly seen in metacarpophalangeal, interphalangeal, and distal interphalangeal joints. It can also occur on the nail borders [7,17]. Gottron’s sign presents as an erythematous macule arranged in a linear pattern on the extremities that undergoes desquamation later. It occurs on the dorsal and lateral aspects of the hands, fingers, extensor surfaces of elbows, knees, and ankles [7,17].

Characteristic skin features are heliotrope rash, nail fold changes, shawl sign, V sign, holster sign, and scalp involvement. Heliotrope rash manifests as a purplish, pruritic, erythematous rash with edema on the upper eyelids mainly. It may also affect the cheeks, nose, and nasolabial folds. The Shawl sign is a violaceous erythematous patch or macule involving the neck, upper back, posterior, and lateral shoulders, whereas the V sign involves the upper chest. Nail fold changes comprise periungual erythema and telangiectasia, hemorrhagic nail fold infarcts, and dystrophy or overgrowth of cuticles. This is known as Keining’s sign. Scalp involvement presents as pruritic scaly plaques which can be misdiagnosed as psoriasis and seborrheic dermatitis [7,17]. Non-scarring alopecia was reported in some patients, especially during a flare of DM. The holster sign is poikiloderma of the hips and lateral thighs. It can be reticulated, linear, or livedoid. It has been found to be highly specific for DM [7]. 

Compatible skin findings include poikiloderma, periorbital edema, and facial swelling. Poikiloderma presents as hypopigmented or hyperpigmented telangiectasia and atrophy, invoking the upper chest, upper arms, thighs, and buttocks [7,17]. It is also called poikiloderma atrophicans vasculare or poikilodermatomyositis [7]. Similar skin findings occur in mycosis fungoides and chronic radiodermatitis. So, a punch biopsy is recommended to rule out other conditions [17].

Less common skin findings are cutaneous vasculitis manifestations and calcinosis cutis. Cutaneous vasculitis presents as vesiculobullous, ulcerative, and necrotic lesions. Cutaneous vasculitis is seen in a majority of patients with JDM [7,17]. Calcinosis cutis is characterized by cutaneous or subcutaneous deposition of calcium. It manifests as nodules on the elbows, knees, and buttocks. It is seen in 70% of JDM patients and 10% of ADDM patients. It is associated with solid neoplasia or hematologic malignancy [17].

Rare skin features are mechanics' hands, flagellate erythema, panniculitis, mucinosis, inverse Gorton’s papules, erythroderma, and oral mucosal changes. Mechanics' hands share features of contact dermatitis such as hyperkeratosis, scaling, and fissuring of the lateral fingers and palms. On rare occasions, flagellate erythema has been found in DM patients. Multiple linear erythematous lesions, resembling whiplash marks on the skin on the back, lateral chest, and upper buttocks, are found. Panniculitis presents as painful erythematous nodules on the upper thighs, buttocks, arms, and abdomen [7,17].

The deck chair sign may be the first skin finding, manifesting as erythematous lesions sparing skin folds [7]. Erythematous papules and plaques in a reticular fashion are seen in mucinosis. Oral mucosal changes include gingival telangiectasia, erosions, ulceration, hyposalivation, and leukoplakia [7,17]. Ghali et al. reported that gingival telangiectasia is the most common oral finding and an important diagnostic marker in JDM [19]. Bullous lesions in DM have only been mentioned infrequently in the literature [17].

Patients with DM may present with one or a constellation of cutaneous symptoms. It may precede or follow myositis; it need not occur in parallel to muscle manifestations [7].

Systemic Manifestations

DM presents with acute or subacute onset of symmetrical proximal muscle weakness. Patients may also present with dysphonia, dysphagia, and symptoms of aspiration due to weakness of striated muscles of the pharynx and esophagus [7,17].

Different organ systems can be involved in DM [7]. ILD can be seen in 40% of patients with DM during the clinical course of the disease. Patients with anti-Jo antibodies have been reported to have a better ILD prognosis. Clinical features of ILD include dyspnea on extortion, coughing, and impaired exercise tolerance. The following are the three different clinical courses that have been described: acute and severe involvement; chronic, slowly progressing symptoms; an asymptomatic disease with evidence of lung damage on imaging. Muscular symptoms occur before lung disease, however, this is not always the case. Pulmonary function tests are of the restrictive type (FVC/TLC<80% predicted age). The most common treatment for ILD is corticosteroids. FVC has been shown to improve after a year of continuous corticosteroid treatment. Azathioprine is often utilized as a corticosteroid-sparing adjuvant. Mycophenolate mofetil and rituximab are two other treatment options [17].

Histologically, two distinct subtypes of muscle involvement have been identified. Capillary loss, C5b-C9 complement deposition on the capillary endothelium, endothelial microtubular inclusions in the muscle along with perivascular infiltrates of lymphocytes as well as macrophages in the perimysium are consistent with the myovasculopathy type of DM. Myofiber necrosis, perifascicular regeneration, and little T-cell lymphocytic inflammatory infiltrates are consistent with the immune-mediated necrotizing pattern of DM [17]. 

Cardiac manifestations include subclinical diastolic dysfunction, myocarditis, myocardial fibrosis, congestive cardiac failure, and arrhythmia [7,17].

Role of autoantibodies in dermatomyositis

Dermatomyositis autoantibodies can be of two types. (1) Myositis-associated autoantibodies (MAA): anti-PM Scl, anti-U1, anti-U2, anti U3-RNP, anti-Hu anti-SSA/anti-Ro and anti-Sjogren's syndrome B (anti-SSB)/anti-La autoantibodies [17]. (2) Myositis-specific autoantibodies (MSA): anti-Mi-2, anti-melanoma differentiation-associated protein-5 (anti-MDA-5), anti-nuclear matrix protein-2 (anti-NXP-2),anti -TIF-1 and anti-small ubiquitin-like modifier activating enzyme (anti-SAE)-1/2 [7,14,15,17,18].

Myositis Specific Autoantibodies

It is now widely accepted that DM is linked to a fascinating array of myositis-specific autoantibodies [17,18]. These MSAs play an important role in the diagnosis and classification of DM as each MSA is associated with characteristic clinical features, organ pathology, HLA association, and micro RNA profiles [18]. MSAs are specific antibodies against proteins present in the cytoplasm or nucleus of cells [20]. MSAs can be detected several months before the development of clinical manifestations. The serum levels may be related to the severity of the condition. Decreasing levels of MSAs indicate remission, hence they can act as prognostic markers [20]. More than 60% of patients with dermatomyositis have detectable levels of MSAs [18]. 

Anti-Mi-2 autoantibodies: Anti-Mi-2 antibodies are autoantibodies targeted against nuclear DNA helices [7]. The Mi-2 antigen is a constituent of the nucleosome remodelling deacetylase complex [14]. Anti-Mi-2 antibodies are detected in similar numbers in DM patients all over the world: 2-30% in Asia, 8% in Brazil, and 10-21% in Europe and the USA. A meta-analysis of various studies showed that the prevalence of anti-Mi-2 antibodies is 9% in DM patients with a 95%CI of 9-14% [18].

Patients with anti-Mi-2 antibodies have a decreased risk of ILD and a low risk of malignancy [7,14]. In Mi-2DM, proximal muscle weakness is associated with higher levels of creatine kinase (CK) and lactate dehydrogenase (LDH) in both adults and children [7,14]. Common skin findings include Gottron’s papules and signs, heliotrope rash, V sign, shawl sign, cuticular overgrowth, and flagellate erythema [7,14]. Japanese patients with anti-Mi-2 positive DM had a higher prevalence of punctate perionychium hemorrhages when compared to those with anti-MDA-5 and anti-TIF-1- 𝝲 autoantibodies. A cohort study conducted in the USA showed a positive association between anti-Mi-2 antibodies and UV exposure in women [14].

Anti-Mi-2 antibodies are present in four to ten percent of JDM patients, with prognosis and clinical features similar to those seen in adults and children [7]. Mi-2 has a good prognosis and responds well to treatment [7,14]. Rituximab has recently emerged as a promising therapy option for patients with anti-Mi-2 antibodies, with findings from the Rituximab in Myositis (RIM) trial revealing a significant improvement in interferon chemokine score and clinical illness [14].

Anti-MDA-5 autoantibodies: MDA-5, also known as Ifih1 and Helicard, is a cytoplasmic RNA helicase in the RIG-1 (retinoic acid-inducible gene-1) family. Two caspase recruitment domains and a DExD/H-box helices domain are present in both RIG-1 and MDA-5. They play an important role in the innate immune system during RNA viral infections. Different ds-RNAs are recognized by these molecules. Picornaviruses and longer double-stranded RNAs are detected by MDA-5 rather than RIG-1 [21]. Anti-MDA-5 antibodies are discovered in a substantially higher percentage of Asian DM patients, 11-37% compared to 0-13% in European and American patients [18]. Patients with anti-MDA-5 antibodies have a higher risk of ILD, arthralgia, arthritis, and decreased creatine phosphokinase (CPK) [14]. Higher levels of anti-MDA-5 are seen in rapidly progressive ILD [7,14]. This autoantibody has a unique association with higher serum ferritin levels in rapidly progressive ILD. It can serve as an alternative to anti-MDA-5 titer levels while monitoring response to treatment and the status of pulmonary disease [14].

Anti-MDA-5 DM has been linked to an increased risk of severe cutaneous findings such as Gottron’s pauses, inverse Gottron’s papules, panniculitis, mechanics' hands, and heliotrope rash [7,14]. These patients can also present with a fever of unknown origin [14]. In the absence of RP-ILD or pre-existing cardiovascular disease, anti-MDA-5 patients have experienced severe myocardial dysfunction leading to third-degree heart block [14].

The third most commonly detected antibody in JDM is anti-MDa-5 autoantibodies, after anti-TIF-1transcription intermediary factor 1 anti-NXP-2. The risk of developing ILD in children is similar to that of adult patients with anti-MDA-5 antibodies. Also, there is a decreased risk of muscle involvement and arthritis in JDM patients [7].

Anti-NXP-2 autoantibodies: anti-NXP-2, previously known as anti-MJ, was discovered in JDM patients [14,15,18]. It plays an important role in the p53 regulation [14,18]. The prevalence of anti-NXP-2 antibodies varies from 3% to 30% in the United States and Europe and barely 2% to 4% in Japan [18].

Anti-NXP-2 antibody-positive patients had fewer severe cutaneous manifestations and more pronounced muscle complaints [15]. Studies have shown that anti-NXP-2 is associated with severe muscle disease, including dysphagia, myalgias, and classic cutaneous findings. Weakness of distal muscles is also linked with this subset of DM [7,14]. Up to 35% of patients are known to have peripheral edema. Calcinosis and distal ulcerations are seen occasionally [7]. Many studies have confirmed the association between anti-NXP-2 antibodies and malignancy [15].

It is found that this is the second most common autoantibody in JDM in most of the regions [14,18]. Anti-NXP-2 positive JDM patients are affected with severe myopathy, resulting in significant functional impairment and the development of contractures. Calcinosis cutis is seen in more than 40% of patients with this antibody [7]. It was found that there was a greater prevalence of gastrointestinal bleeding and ulcers due to vasculopathy-induced muscle ischemia [7,14]. Anti-NXP-2 antibodies conferred an increased risk of malignancy in ADDM patients [7].

Anti-TIF-γ autoantibody: It is the most common antibody seen in DM patients [14]. They belong to the family of TIF-1, which regulates transcription, tumor suppression, and DNA repair [14]. The prevalence of anti-TIF-1 antibodies ranges from 8% to 14% in the Unites States and Europe, but only 7-14% in Japan [18].

It has been demonstrated that anti-TIF-1-γ antibodies are associated positively with malignancy and negatively with calcinosis cutis, Raynaud's phenomenon, arthralgia, and ILD [14,15]. Younger adults had a decreased risk of neoplasia [15]. Anti-TIF-1-γ positive patients had widespread skin lesions such as poikiloderma, psoriasis-like lesions, and red-on-white telangiectatic patches [15,16]. Although there is a trend for increasing malignancy in the patient population, the prevalence of cancer in the US population is considerably lower [14]. In contrast to adults, anti-TIF-1-γ in children is not associated with malignancy [7].

Anti-SAE: This is one of the most recently discovered MSAs by Betteridge et al. and was first reported in 2007 [15,22]. The prevalence of anti-SAE antibodies varies from 5% to 10% in the USA and Europe, in contrast to 2-3% in Asia [18].

At the time of diagnosis, patients who tested positive for these antibodies had extensive skin involvement and mild myopathic symptoms [7,15]. Clinical evidence of myositis and systemic manifestations occurred in the majority of patients. Notably, more than 75% of patients had dysphagia and more than 80% exhibited systemic symptoms such as fever, weight loss, and elevated inflammatory markers [15]. This subset of DM is strongly associated with HLA DRB1*04-DQA1*03-DQB1*03 [7,15]. Mild ILD was seen in 18% of patients [14,15]. Anti-SAE antibodies, in particular, have been observed to predict hydroxychloroquine medication eruptions [7]. 

Anti-SAE JDM affects only a small percentage of JDM in children (6-8% in European cohorts and 2% in Asian cohorts) and is characterized by severe cutaneous involvement and moderate muscle involvement [7].

Other Novel Autoantibodies

Skeletal and cardiac muscles express four-and-a-half LIM domain (FHL1) proteins. Studies have shown that 25% of patients with IIM had anti-FHL1 autoantibodies detected in their sera. Anti-FHL-1 antibodies are associated with muscle atrophy, dysphagia, muscle fiber damage, and vasculitis [15].

Variants of dermatomyositis

Clinical Amyopathic Dermatomyositis

Patients with clinical amyopathic dermatomyositis (CAMD) have no clinical or laboratory signs of muscle disease, as well as clinically apparent muscular features as demonstrated by MRI, electromyography, or muscle biopsy with normal muscle enzyme levels for at least six months; 20% of DM patients are found to have no muscle involvement or subclinical muscular manifestations [17,23]. A study conducted by Ye et al. reported that CAMD patients are more prone to developing rapidly progressive ILD, which has a poor prognosis [24]. A systematic review of CADM patients conducted in 2006 revealed that 14% of 301 CADM patients had an associated malignancy, with the most common being nasopharyngeal carcinoma and breast cancer [17,23]. As a result, it appears that sufficient malignancy screening might be beneficial in CADM patients as well [17]. A subgroup of CADM patients may develop classic DM six months after initial presentation. Retrospective studies have shown that CADM patients have typical skin findings like classic DM without muscle weakness or elevated CK levels. Furthermore, these patients do not appear to be defined by a single autoantibody but can have a wide range of DM-specific autoantibodies [23].

Juvenile Dermatomyositis

JDM is a chronic autoimmune disorder that affects children and adolescents, most commonly seen in the 4-20 age group, with a female predominance [17,25]. Skin findings in JDM include heliotrope rash, Gottron’s papules, shawl sign, V sign on the upper chest, facial rash, and oral ulcers [17,25]. Other nonspecific findings in children are fever, malaise, and weight loss [17].

Calcinosis cutis is a type of dystrophic calcification characterized by cutaneous or subcutaneous deposition and can extend to the muscle as well [17,25]. It is commonly seen on the elbows, digits, knees, and buttocks [25]. A younger age of onset and a prolonged duration of disease are risk factors for the development of calcinosis [25]. Complications of calcinosis are skin ulceration, nerve compressions, and contractures of joints. Treatment options include diltiazem, a calcium channel blocker; minocycline (acts as a calcium chelator); bisphosphonates (inhibits pro-inflammatory cytokine formation), rituximab, and TNF-inhibitors like infliximab [17]. Skin ulceration, a severe skin complication of JDM, has been reported in 20% of patients [25]. Unlike ADDM, JDM has not been linked to malignancy, and the incidence of malignancy in children with JDM has only been described in case reports [17].

Proximal muscle weakness is one of the cardinal features of JDM, presenting with symptoms such as difficulty in climbing up or downstairs, combing hair, choking while drinking fluids, or voice changes. Nailfold changes in JDM are dilatation, occlusion, hemorrhage, end-row capillary loops, and increased tortuosity. ILD and pneumothorax are pulmonary manifestations. Cardiovascular involvement may be in the form of myocarditis, pericarditis, congestive cardiac failure, and conduction defects. Gastrointestinal findings include dysphagia, gastrointestinal reflux, bowel dysmotility, delayed gastric emptying, gastroparesis, vasculitis of the stomach, ulceration, hemorrhage, perforation, and other features of the liver, small intestine, colon, and rectal disorders. Arthritis is also a common symptom of JDM [25].

Wong Type of Dermatomyositis

Wong type DM is a rare type of DM characterized by the presence of DM with cutaneous symptoms of pityriasis rubra pilaris (PRP). It is named after Wong, who described a series of 11 patients with DM and specific skin lesions on the back of their hands consisting of hyperkeratotic, erythematous follicular confluent papules distributed in a linear way over bony prominences on the neck and back. Heliotrope rash and Gottron’s papules are more common, but palmar-plantar keratoderma (PPK) has been reported infrequently. Although few Wong type of Dermatomyositis (WDTM) patients without muscular symptoms have been described, muscular symptoms have been reported clinically. The appearance of cutaneous and muscular involvement has a varied temporal relationship. The link between WDTM and neoplasia is not completely understood. Twelve cases of paraneoplastic DM were documented in Wong’s collection. However, it was unclear whether the neoplasms were related to traditional DM or WTDM [17]. 

Dermatomyositis as a Paraneoplastic Syndrome

Paraneoplastic syndromes are a group of cancer-related disorders that affect an organ or tissue but arise in a distant region from the primary or metastases. Dermatomyositis can occur in conjunction with cancer as a paraneoplastic condition [26]. In up to 20% of patients, DM manifests as a paraneoplastic syndrome linked to a variety of cancers, including breast, prostate, ovarian, lung, nasopharyngeal, and colorectal cancers, as well as non-Hodgkin's lymphomas [27].

It was recently shown that tumors from anti-TIF-1-positive paraneoplastic patients have an increased number of genetic abnormalities, such as mutations and loss of heterozygosity (LOH) in TIF-1 genes [28]. LOH is the most common route for a mutant allele to be lost in human cancer, and it is important for tumor immune-editing because tumor cells with neo-antigen-producing mutations may be removed by the immune system and replaced with tumor cells with LOH in that area (without the antigenic mutation). These changes can elicit an immune response, but they can also allow tumor cells to evade clearance [28].

For patients with paraneoplastic dermatomyositis (pDM), a link between pDM and anti-TIF-1c (p155) autoantibodies has been found, with a sensitivity of 78%, and anti-p155-1 was found to be negative in around 90% of patients without pDM. However, as anti-TIF-1c autoantibodies are also discovered in juvenile DM without a cancer link, it appears that older age is the decisive factor. Anti-Mi-2 autoantibodies have also been seen in up to 21% of people with pDM [29].

In contrast to this normal presentation of DM, patients may experience rapid progression of symptoms over a considerably shorter period of time, with the majority of patients reporting cutaneous signs followed by rapid muscle degeneration [30]. Patients under 60 years of age with abrupt onset or substantial skin involvement and/or severe myositis, recurring disease, and high CK levels should be investigated for developing and/or progressing malignancy/pDM as soon as possible, preferably within three years of DM diagnosis [29]. Although whole-body positron emission tomography/computed tomography (PET/CT) is a very useful technology for detecting malignancy, its value in screening pDM individuals for concealed malignancy does not appear to be superior to CT screening [27]. Early detection of DM as a paraneoplastic illness allows for the commencement of systemic therapy for symptom control as well as treatment of the underlying tumor, which may aid in symptom control [30].

Association Between Dermatomyositis-Specific Antibodies and Risk of Malignancy

Several epidemiological studies have proven the link between cancer and myositis, and patients with DM have the highest risk [31]. In comparison to the general population, DM is linked to a sixfold increased risk of cancer, especially in the first two years after diagnosis [32]. Malignant illness can arise before the development of DM, concurrently, or after the onset of DM [33]. Breast cancer, lung cancer, and colorectal cancer are the three most prevalent cancers diagnosed in people in Western culture, whereas nasopharyngeal cancer is one of the most common diseases in southern China and Southeast Asia [34]. Cancers such as ovarian, pancreas, and gastrointestinal cancers as well as non-Hodgkin’s lymphoma are also significantly linked to DM [35].

One idea for the link between dermatomyositis and cancer is that tumors produce oncoproteins that trigger an immunological response, and autoantibodies are deposited in the skin and muscle, which may contain comparable antigens. Myositis-specific antibodies are found in about 30% of DM patients [36]. Melanoma differentiation-associated protein 5 (anti-MDA-5), TIF-1, and NXP-2 cancer (also known as MORC3) are three novel DM-specific autoantibodies that may be useful in predicting the risk of developing cancer [34].

Breast cancer: The precise link between breast cancer and DM has yet to be established. There is no defined guideline for oncologists addressing these patients. DM is most typically associated with invasive ductal carcinoma and appears to be connected with more advanced stages (stages III and IV) of breast cancer. Patients with breast cancer experiencing symptoms of dermatomyositis should be evaluated with muscle enzymes, MRI, electromyography, and a muscle biopsy for tissue confirmation. MSAs including Mi-2, TIF-1, MDA-5, Jo-1, and PM/Scl are not always positive, but they can help with diagnosis and subtyping of dermatomyositis [37].

Colorectal cancer: Despite a diverse array of possibilities, the underlying pathophysiology of the link between DM and cancer, particularly in the case of colorectal cancer, is not entirely understood. Serum autoantibodies (such as antinuclear antibodies and anti-Jo1) were found in 40.7%. The neoplastic disease can occur before, during, or after the diagnosis of DM. Adenocarcinomas are the most common histological type of colon cancer in DM individuals. The emergence of cancer within a year of the first diagnosis of DM suggests that colorectal neoplasia was most likely occult at the time of diagnosis. Hence, screening for underlying neoplasia should be done for these patients, including colonoscopy [38].

Ovarian carcinoma: Ovarian carcinoma is substantially more common in people with DM (14%) than in the general population (4%), according to a major study. The most common histologic subtype was serous ovarian cancer, which was detected at stage III or IV. Ovarian cancer is extremely difficult to detect during routine check-ups and/or periodic ultrasounds or CT scans. Early identification may be aided by screening tests such as serum CA-125 and variations on the currently available monoclonal antibody approach for ovarian cancer. In women of all ages diagnosed with dermatomyositis, clinicians should have a high clinical suspicion for underlying ovarian cancer, especially if an increased anti-p155/140 auto-antibody is present during a specific time period which is two to three years from the onset of dermatomyositis [39,40].

The Role of Antibodies in Assessing the Risk of Malignancy in Patients With Dermatomyositis

The possibility of a link between DM and cancer poses the question of whether the two conditions are related or if the myopathy is a consequence of an immune response to the tumor. Although antibodies to tumor extracts have been observed in numerous patients, this phenomenon can also be seen in cancer patients without DM [41]. Because of this link between DM and cancer, it is critical that DM patients be adequately tested for the presence of an underlying tumor [27].

Antisynthetase (Jo-1), anti-Mi-2, anti-MDA-5, anti-TIF-1, anti-NXP-2, and anti-SAE antibodies are among the disease-specific autoantibodies that can be used to classify the dermatomyositis spectrum [15]. Anti-TIF-1-g and anti-nuclear matrix protein-2 antibodies suggest a greater risk of cancer, while Jo-1 is an MSA that recognizes histidyl-RNA-synthetase and belongs to the group of antibodies against aminoacyl-tRNA synthetases [15] and is an immunological marker for DM that has an increased diagnostic specificity but is only found in 30% of patients [34].

Anti-TIF-1-g antibodies are found in 50-100% of cancer-related DM patients [21]. Anti-TIF proteins are overexpressed in numerous cancer forms, as well as the fact that an autoimmune response to TRIM28 is a common finding in patients with colorectal carcinoma (even without DM), backs up this theory [42]. It is only found in 23-29% of juvenile DM patients [34] and is one of the most commonly found antibodies in juvenile DM, but it is not linked to malignancy, and patients under the age of 40 have no increased risk of malignancy even though they possess the antibody [43].

There is a strong correlation between NXP-2 antibodies and the chance of acquiring cancer, which is frequently and unexpectedly connected with the male gender, although the explanation for this link is unknown [34].

It is well known that cancer risk increases with age in DM patients, and recent research has found a link between cancer incidence and age 60, as well as the presence or absence of anti-NXP-2 and anti-TIF-1. The incidence of cancer is relatively low (2.6%) in individuals over 60 with no anti-TIF-1 or anti-NXP-2 autoantibodies, but it is greater (11%) in patients under 60 with anti-TIF-1 or anti-NXP-2 autoantibodies [34].

Anti-Mi 2 antibody is almost exclusively found in DM patients, particularly those with typical cutaneous DM lesions and no ILD. According to a European study, anti-Mi-2 antibody-positive patients who had the N-terminal portion of the Mi-2 antigen were shown to have a higher risk of cancer. Anti-CADM-140 antibody has been linked to amyopathic DM and acute progressive interstitial pneumonia, however, it has not been linked to the development of cancer [31].

Future of These Antibodies in Diagnosis, Prognosis, and Treatment of Dermatomyositis

MSAs strongly correlate with various disease phenotypes, allowing MSA-based categorization of dermatomyositis subgroups [15]. Furthermore, MSAs have also been used as diagnostic and prognostic indicators [44,45].

Identification of autoantibodies

Autoantibody detection for myositis until recently relied on procedures like immunoprecipitation (IP) tests that were not generally accessible, limiting the antibodies’ value as biomarkers [46]. Therefore, there has been a lot of interest in creating and verifying non-specialized procedures to make autoantibody testing more accessible [47]. Recently, several MSAs/MAAs have been identified and well-characterized, and commercial assays for their detection have become available [48]. A variety of assays are used for the identification of individual MSAs/MAAs, including RNA and protein IP assays, enzyme immunoassay (EIA), line-blot immunoassay (LIA), and Ouchterlony double immunodiffusion (DID). Recently, particle-based multianalyte technology (PMAT) was established as the next-generation multianalyte immunoassay to detect autoantibodies [48]. High concordance between the results of PMAT and LIA or IP assays was reported [48]. Although several of these tests are commercially accessible, the rarity of myositis, particularly juvenile-onset illness, has hindered postmarketing validation, especially within individual laboratories with small sample sizes [48].

When multiplex assays are used, the asking physician must understand which autoantibodies are included in the test because a negative result may necessitate additional testing for a different autoantibody depending on the clinical presentation [48]. Furthermore, no testing methodology is perfect, and false positives occur [48]. Identifying these instances may be aided by knowledge of the related immunofluorescence pattern [48]. The importance of autoantibody titers and quantitative tests in developing accessible assays for myositis-specific autoantibodies should not be underestimated [47]. Unfortunately, there is insufficient evidence to provide specific guidelines for using MSAs to guide clinical care [48]. MSAs can be employed to optimize management methods in individuals with DM if data from larger prospective cohorts and standardized detection techniques are available [48]. However, detection of MSAs and MAAs may be difficult in immunosuppressed patients, reducing the efficacy of these antibodies as biomarkers.

The addition of new antibodies like anti-TIF-1-γ, anti-Mi-2, anti-MJ/NXP-2, anti-SAE, and anti-small ubiquitin-like modifier-1 (SUMO-1) to clinical practice in the future will help in making earlier and more accurate diagnoses and better management of patients [49]. In addition, MSAs will undoubtedly be employed in the future to customize treatment options for DM patients [15]. However, there is currently a scarcity of evidence to justify such a strategy [15,49].

This scarcity of evidence is due, at least in part, to the fact that the current data originates from small studies with varied patient demographics and different autoantibody detection technologies [14]. In addition, clinical phenotypes are most likely the result of a complex interplay between patient variables (such as genetics and environment) and autoimmunity [14]. As a result, instead of universal "antibody phenotypes," it is possible that phenotypes specific to specific clinical strata may emerge [14]. Using a universal "platform" for MSA detection and acquiring data from larger prospective and diverse patient cohorts will enhance our interpretation of the significance of serological data in DM [14].

## Conclusions

Dermatomyositis is a rare autoimmune disease that affects skin, muscles, and multiple visceral organs and causes significant morbidity. Furthermore, because the illness is a paraneoplastic syndrome, proper screening efforts in people with contemporaneous or metachronous malignancy will be possible if the skin signs are recognized. MSAs are an effective tool for distinguishing patients with DM. MSAs are significant auxiliary tools for diagnosis and sub-classification of patients that will lead to specialized clinical care in the future, despite disadvantages such as lack of cross-validation of tests, autoantibody phenotypic overlap, and partial autoantibody penetrance. The diagnostic sensitivity and specificity of tests for various MSAs will not be perfect, just like with any other test. This does not, however, preclude us from continuing to improve techniques for employing the tests as efficiently as feasible. In particular, MSAs are linked to specific combinations of systemic and cutaneous phenotypes, and even in the absence of a standardized autoantibody testing platform, doctors may diagnose dermatomyositis by recognizing these patterns and combinations. Accurate sub-classification of DM patients is crucial for guiding patient care in terms of follow-up, therapy selection, and disease sequelae prediction. More antibody tests are anticipated to make their way into formal schemata for diagnosis and actionable risk assessment in DM as a result of worldwide standardization and more research.
